# A randomized phase 2 clinical trial of phentolamine mesylate eye drops in patients with severe night vision disturbances

**DOI:** 10.1186/s12886-022-02621-6

**Published:** 2022-10-08

**Authors:** Jay Pepose, Mitchell Brigell, Eliot Lazar, Curtis Heisel, Jonah Yousif, Kavon Rahmani, Ajay Kolli, Min Hwang, Cara Mitrano, Audrey Lazar, Konstantinos Charizanis, Mina Sooch, Marguerite McDonald

**Affiliations:** 1 Pepose Vision Institute, St. Louis, MO USA; 2 Department of Ophthalmology and Visual Sciences, Washington University School of Medicine, St. Louis, MO USA; 3 Ocuphire Pharma, Inc., 37000 Grand River Ave., Suite 120, Farmington Hills, MI 48335 USA; 4 elCON Medical, Buffalo, NY USA; 5 Department of Ophthalmology, New York University Langone Medical Center, New York, NY USA

**Keywords:** Night Vision Disturbance, Dim Light Disturbance, Photic Phenomenon, Phentolamine, Halos, Glare, Starburst, NVD, DLD

## Abstract

**Purpose:**

Dim light vision disturbances (DLD) comprise a wide range of symptoms affecting the quality of vision at low illumination including glare, halos, and starbursts. This exploratory study investigated 1.0% phentolamine mesylate ophthalmic solution (PMOS) as a treatment to improve vision and image quality for patients with DLD.

**Methods:**

In this placebo-controlled, randomized, double-masked clinical trial, 24 adult patients with severe DLD were randomized in a 2:1 ratio to receive either one dose of PMOS or placebo. Subjects were eligible if they reported experiencing severe night vision difficulty that was not eliminated by distance spectacle correction and scored ≥0.3 log units below the normal range of contrast sensitivity assessed under mesopic conditions with glare at ≥2 spatial frequencies. Key efficacy outcomes were change from baseline in pupil diameter, contrast sensitivity, and visual acuity. Safety measures including intraocular pressure, conjunctival hyperemia, and systemic effects were also assessed.

**Results:**

Eight subjects were randomized to placebo (63% female; mean age 47 years) and 16 were randomized to PMOS (75% female; mean age 42 years). Mean (SD) pupil diameter of PMOS-treated subjects decreased significantly − 1.3 mm (0 to − 2.8 mm) with *p* < 0.0001. Mean contrast sensitivity with glare in PMOS-treated subjects improved significantly post-treatment at spatial frequencies 3, 6, 12, and 18 cycles per degree (*p* ≤ 0.03). PMOS also demonstrated improvements in the numbers of letters read for mesopic and photopic, high- and low-contrast visual acuity (LCVA). Importantly, a statistically greater proportion of PMOS-treated eyes registered mesopic LCVA 5 letter (69% vs. 31%, *p* = 0.029) and 10 letter (34% vs. 6%, *p* = 0.04) improvement, with a trend at 15 letters (19% vs. 0%, *p* = 0.16). PMOS was well tolerated with the only reported side effect being a mild increase in conjunctival hyperemia.

**Conclusion:**

PMOS was well tolerated and effectively reduced pupil size with improvements in contrast sensitivity and visual acuity in adults with severe DLD. Future Phase 3 studies should be conducted to further evaluate its potential to treat DLD.

**Trial registration:**

The trial registration number is NCT04004507 (02/07/2019). Retrospectively registered.

## Introduction

Night vision or dim light vision disturbances (DLD) encompass photic phenomena, including glare, halo, and starbursts, which can result from ocular aberrations, ocular scatter, and superimposed retinal images in the case of multifocal intraocular lenses [1]. Millions of patients who suffer from DLD have difficulty with night vision due to the physiologic dilation of the pupil that occurs in dim lighting conditions. A dilated pupil allows skewed rays of light to enter the eye from the periphery. This increases higher-order ocular aberrations, which can contribute to photic phenomena. DLD are more common among those with elevated levels of higher- or lower-order aberrations, including irregular corneal astigmatism, keratoconus, residual refractive error, prior refractive surgery (e.g. laser-assisted in situ keratomileusis [LASIK], photorefractive keratectomy [PRK], and radial keratotomy [RK]), multifocal or depth of focus intraocular lenses (IOL), or increased ocular scatter (e.g. cataract, dry eye, and corneal scars) [1–6]. DLD can have a significant impact on quality of life. For example, after photorefractive surgery, approximately 30% of individuals experience worsening in their driving capabilities, particularly prior to wavefront guided or optimized treatments and larger optical zone ablations [1].

One manner in which DLD can be mitigated is through miosis, in which a smaller pupil blocks aberrant peripheral light entry [7]. Pupil size is controlled by two muscles: the iris dilator muscle (controlled by the adrenergic nervous system) and the iris sphincter muscle (controlled by the cholinergic nervous system). Since the iris dilator muscle predominantly responds to alpha-1 adrenergic receptors, alpha-1 antagonists inhibit the iris dilator muscle and induce a desired miotic effect [8].

There are currently no FDA-approved pharmacological therapies for DLD. Previous approaches to mitigating DLD have included off-label use of the topical miotic agents pilocarpine and brimonidine. However, side effects associated with these miotic agents have limited their widespread clinical use as a means of reducing DLD. Specifically, pilocarpine causes a myopic shift in pre-presbyopic patients and frequently causes headaches and ciliary muscle spasm, while repeated use of brimonidine can lead to tachyphylaxis, rebound mydriasis and allergic follicular conjunctivitis [9, 10]. In addition, reports of retinal tears and detachment and vitreofoveal traction have been reported in association with topical pilocarpine use for presbyopia [11–13]. A non-selective alpha-1 and alpha-2 adrenergic antagonist miotic agent, phentolamine mesylate, is a potential alternative that may be better tolerated than pilocarpine and brimonidine. More specifically, pilocarpine and other cholinergic agents’ activation of the iris sphincter and ciliary muscle may be associated with side effects such as browache, headache and increased risk of retinal tears or detachment in some individuals due to shallowing of the anterior chamber causing vitreous traction. These cholinergic miotic side effects are obviated by the use of phentolamine mesylate, where the mechanism of action is specific to relaxation of the iris dilator with no impact on the ciliary muscle or shallowing of the anterior chamber or increased vitreous traction. A proprietary eye drop formulation, phentolamine mesylate ophthalmic solution (PMOS), is under clinical development for DLD and other pupil modulation indications.

Decreased mesopic distance low contrast visual acuity (mLCVA) and mesopic contrast sensitivity (CS) are key indicators of vision loss in dim light, a surrogate for DLD [14]. There is low concordance between photopic and mesopic CS, and photopic (bright light) CS may be deemed a less relevant surrogate for certain real-life situations, such as driving at night [14, 15]. In fact, lower mesopic visual function has been associated with worse nighttime driving performance [16]. Moreover, compared to photopic CS, mesopic CS has been found to be superior in detecting between-person differences in visual function [17]. Accordingly, a two-patch (i.e. 0.3 log unit increase) in mesopic CS has been used in prior literature as an indicator of clinically significant improvement for those with DLD [18].

Results from a randomized clinical trial assessing the efficacy and safety of 1.0% PMOS to treat patients with DLD are presented here. It is hypothesized that a reduction of pupil size with a single topical dose of PMOS (1) improves mesopic CS and mLCVA, (2) decreases wavefront aberrometry measures, and (3) exhibits a tolerable safety profile. If PMOS demonstrates efficacy and safety, then it may prove to be a viable treatment option to improve visual function in patients with DLD.

## Methods

### Patient selection

Subjects included in the single-center study were individuals 18 years of age or older who reported experiencing severe night vision difficulty, scored ≥0.3 log units below the normal range of contrast sensitivity assessed under mesopic conditions with glare at ≥2 spatial frequencies, and demonstrated a two-line improvement in low contrast visual acuity (LCVA) in dim light during illumination of the contralateral eye at screening. Monocular contrast sensitivity was measured using the Optec 6500 (Stereo Optical, Chicago, IL) linear sine-wave grating charts viewed through distance corrected lenses. The stimuli were sine-wave gratings of 1.5, 3, 6, 12, and 18 cycles per degree (cpd) in circular patches (diameter: 1.7°). For each spatial frequency, nine stimuli in 0.15 log contrast decrements were presented on an equiluminant grey background. The gratings were shown at one of 3 orientations, vertical, and tilted by 15° clockwise or counter-clockwise and subjects made forced-choice responses regarding the orientation of the gratings [19].

Exclusion criteria included untreated cataracts, current contact lens use, recent (within 5 weeks) refractive surgery (e.g. LASIK or PRK) or IOL insertion, systemic hypotension, a history of heart rate abnormalities, recent administration of any investigational drug (within 30 days), recent (within 7 days) use of any eye drop with a pharmacologic effect on the pupil, current use of any systemic alpha-adrenergic antagonists, known local or systemic hypersensitivity to adrenergic antagonists, or current pregnancy.

The following article reports the results of a drug intervention on human participants. This single center investigator-initiated trial was retrospectively registered in the clinicaltrials.gov database (NCT04004507) on 02/07/2019, with the dates of recruitment to follow-up spanning from Aug to Oct 2007. However, the retrospective registration did not influence the integrity of the study or the opportunity for subjects to participate. All methods in the described trial were performed in accordance with the Declaration of Helsinki and other relevant guidelines and regulations. The study was approved by the WIRB-Copernicus Group Institutional Review Board (WIRB Study No.: 1090116). All subjects provided written informed consent to participate in the trial and were in good general health. The trial was conducted at the practice of Ophthalmic Consultants of Long Island in Lynwood, New York.

### Randomization and treatment

Subjects were randomized in a 2:1 ratio to the phentolamine mesylate and placebo treatment group, respectively. Patients in the placebo group received one drop of polyquad lubricant eye drop (Tears Naturale II^Ⓡ,^ Alcon, Fort Worth, TX) in each eye. Patients in the treatment group received one drop of 1.0% phentolamine mesylate prepared in Tears Naturale II® vehicle (PMOS) in each eye. This was a double-masked study, where subjects and the investigator were both masked to the treatment regimen. One investigator conducted all of the slit lamp examinations at this single site study.

### Efficacy and safety measurements

The pupil diameter of both eyes was measured in a darkened room at screening, pre-treatment, and 2–3 hrs post-treatment. Subjects were given 2–3 minutes to adjust to room lighting prior to each evaluation. Measurements of pupil diameter were performed with the NPi-200 pupilometer (Neuroptics, Irvine, CA).

Distance corrected monocular high contrast visual acuity was measured under photopic and mesopic conditions for each eye at pre-treatment and at 2–3 hrs post-treatment. Tests were performed in a darkened room using the Optec 6500 instrument (Stereo Optical, Chicago, IL) with distance setting at “far” and with the light setting at “day” for photopic conditions and “night” for mesopic conditions. Forced choice letter-by-letter scoring was used, and the total number of correct letters was recorded.

Monocular contrast sensitivity (CS) was measured using the Optec 6500 (Stereo Optical, Chicago, IL) under mesopic (3 cd/m^2^) and photopic (85 cd/m^2^) conditions with and without additional glare light (1 Lux for mesopic conditions and 10 Lux for day glare testing). The stimuli were sine-wave gratings of 1.5, 3, 6, 12, and 18 cpd in circular patches (diameter: 1.7°). For each spatial frequency, nine stimuli in 0.15 log contrast decrements were presented on an equiluminant grey background. The gratings were shown at one of 3 orientations, vertical, and tilted by 15° clockwise or counter-clockwise and subjects made forced-choice responses regarding the orientation of the gratings. The test was conducted using distance refraction. The test was stopped following two consecutive incorrect responses, and the contrast of the last correctly identified stimulus defined the contrast sensitivity for that spatial frequency [19].

Best-corrected low contrast distance visual acuity was measured for each eye under photopic and mesopic conditions at pre-treatment and 2–3 hrs post-treatment. The tests were performed in a lit (for photopic conditions) and unlit (for mesopic) room using the Precision Vision illuminated box with 5% translucent Contrast chart (#2186) at 4 m. Forced choice letter-by-letter scoring was used, and the total number of correct letters was recorded (42 letters or 9 lines read was equivalent to 20/20 low contrast visual acuity). Best-corrected high contrast distance visual acuity was also measured in photopic and mesopic conditions at pre-treatment and 2–3 hrs post-treatment.

Monocular wavefront (WF) analysis was performed in an unlit room at pre-treatment and at 2–3 hrs post-treatment using a VISX CustomVue™ aberrometer. Measurements taken included WF diameter, total WF root mean square error (RMS error), as well as higher-order RMS error.

A dilated direct ophthalmoscopic examination was performed at screening and at 2–3 hrs post-treatment, with any abnormal findings recorded. In addition, slit lamp evaluation of both eyes was performed to detect any corneal edema, corneal vascularization, corneal staining, limbal hyperemia, bulbar hyperemia, palpebral conjunctival hyperemia, palpebral conjunctival papillae, or palpebral conjunctival staining, using a scale of 0 (no pathology) to 4 (most pathology).

Conjunctival hyperemia was assessed and recorded on a scale of 0 (none) to 100 (worst) on a 100 mm visual analog scale (a modification of the Cornea and Contact Lens Research Unit [CCLRU] grading scale) at pre-treatment and 2–3 hrs post-treatment. All medications concomitantly administered to subjects at screening and at all subsequent visits were recorded. Subjects were asked to subjectively evaluate their night vision at 2–3 hrs post-treatment. Response options included ‘much better’, ‘better’, ‘the same’, ‘worse’, or ‘much worse’.

Intraocular pressure (IOP) was measured at screening and 2–3 hrs post-treatment using applanation tonometry. Heart rate (HR) and blood pressure (BP) were measured at screening, pre-treatment, and 2 hr post-treatment.

### Statistical analyses

All statistical tests were two-sided and used a significance threshold of alpha = 0.05. The sample size was intended to have sufficient power to prove statistical significance for a 0.3 log mean improvement in contrast sensitivity at any spatial frequency between treatment groups at 2 h assuming a standard deviation (SD) of 0.25 with *p* = 0.05. Categorical demographic variables were compared using Fisher’s exact test to assess for sufficient randomization. Continuous demographic variables were compared using two-sample t-tests. Efficacy outcomes were described, and differences between the treatment and placebo group were evaluated using two-sided t-tests of means assuming equal variances. Differences in incidence of value change above or below a given threshold value between treatment groups were tested for statistical significance using two-sided Fisher’s exact tests. For parameters measured in both the right and left eye, each eye was treated independently and analyzed separately. Calculation of 95% confidence intervals (CI) was performed using a normal approximation to the binomial distribution.

## Results

Twenty-four patients were successfully enrolled in the study. Eight patients were randomized to the placebo group and 16 were randomized to the treatment group. Demographic and baseline characteristics of all patients are shown in Table 1. There were no statistically significant differences in baseline characteristics between those randomized to the placebo vs. treatment group.

**Table 1:** Demographic variables and baseline measurements

Demographic variable	Placebo	1.0% Phentolamine	***P***-value^**a**^
Sample Size	8 (100%)	16 (100%)	–
Female (%)	5 (63%)	12 (75%)	.68
Mean age (yrs ± STDEV)	47.4 ± 13.5	42.1 ± 14.6	.40
**Night vision complaints**			
Halos	7 (87.5%)	9 (56.3%)	.35
Glare Sensitivity	7 (87.5%)	15 (93.8%)	1.0
Starbursts	5 (62.5%)	11 (68.8%)	1.0
Depth Perception	5 (62.5%)	11 (68.8%)	1.0
Other Concerns	0 (0%)	0 (0%)	NA
**Intraocular pressure**			
Right Eye (mm Hg ± STDEV)	13.8 ± 1.5	13.8 ± 2.7	NA
Left Eye (mm Hg ± STDEV)	13.4 ± 1.3	13.9 ± 1.4	.41
**Blood pressure**			
Systolic (mm Hg ± STDEV)	124.8 ± 13.2	122.8 ± 11.2	.70
Diastolic (mm Hg ± STDEV)	79.8 ± 8.0	81.1 ± 8.7	.73
**Prior vision surgery**			
LASIK, n (%)	2 (25.0%)	1 (6.3%)	.25
PRK, n (%)	0 (0%)	0 (0%)	NA
RK, n (%)	1 (12.5%)	0 (0%)	.33
**Pupil diameter**			
Pre-illumination (mm ± STDEV)	6.6 ± 1.0	6.1 ± 1.7	.52
Illuminated (mm ± STDEV)	4.9 ± 0.8	4.4 ± 0.6	.10
**Low contrast visual acuity**^**b**^			
Pre-illumination (letters ± STDEV)	18 ± 9.4	21 ± 6.7	.46
Contralateral- illuminated (letters ± STDEV)	30 ± 8.4	33 ± 5.4	.30

*Abbreviations*: *STDEV* Standard Deviation, *PRK* Photorefractive keratectomy, *RK* Radial Keratotomy

^a^*p*-values are from two-tailed, two-sampled t-tests for continuous variables and Fisher Exact tests for count variables

^b^18 letters read is equivalent to 20/63. 30 letters read is equivalent to 20/40 + 2

### Pupil diameter

Mean pupil diameter of placebo-treated subjects did not significantly change between pre-treatment and post-treatment (*n* = 16 eyes, mean change = − 0.2 ± 0.5 mm, *p* = 0.08). In contrast, mean pupil diameter of PMOS-treated subjects decreased significantly (*n* = 32 eyes, mean change (SD) = − 1.3 mm (0 to − 2.8 mm), *p* < 0.0001). The difference in mean change between treatment groups was also statistically significant (mean change = 1.1 mm, *p* < 0.0001). In subjects with baseline pupil diameters equal to and above 6 mm, subjects treated with PMOS had a mean change of − 1.48 mm (− 20.4% change) in PD, compared to − 0.38 mm (− 5.2% change) when treated with placebo (*p* < 0.0003).

### Contrast sensitivity

Prior to treatment, mean CS with glare was significantly lower in placebo-treated subjects compared to PMOS-treated subjects at 1.5 cpd (0.75 versus 0.92 log units; *p* = 0.03) and 12 cpd (0.12 versus 0.24 log units; *p* = 0.04). Mean pre-treatment CS with glare was not statistically different between groups at other frequencies tested.

**Fig. 1:**
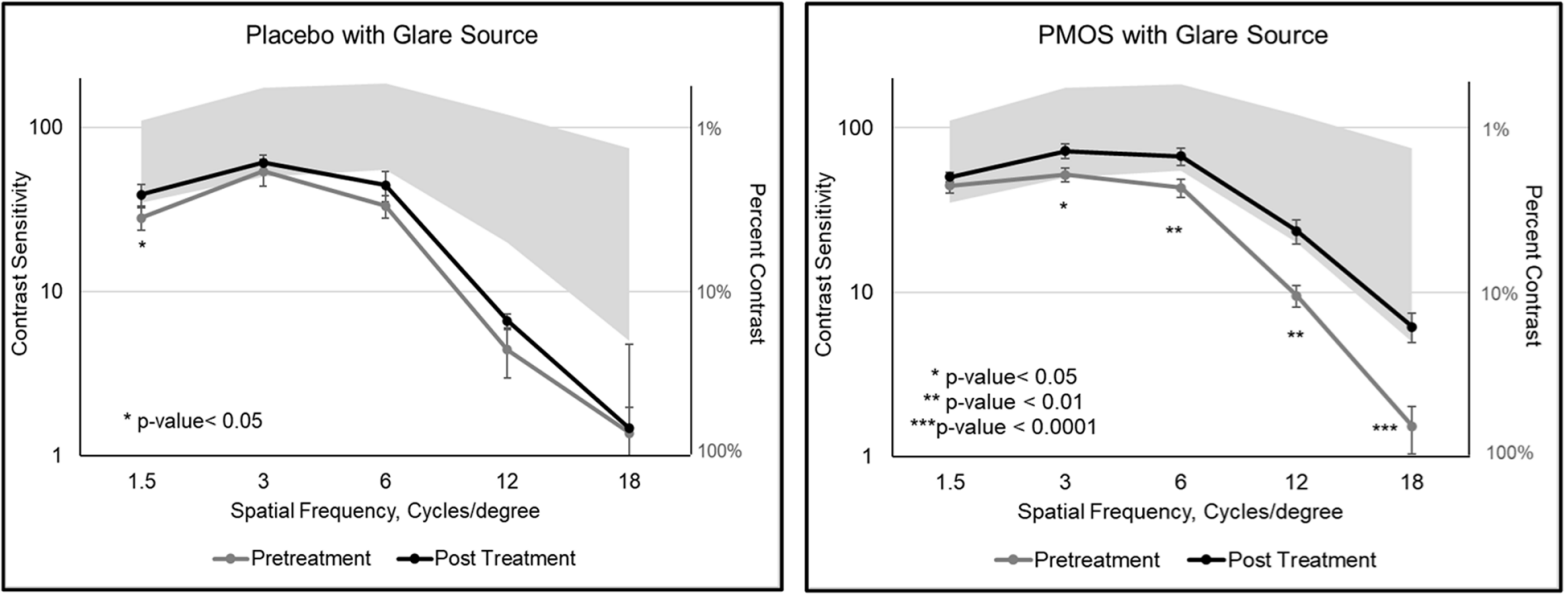
Mean contrast sensitivities with glare before and after treatment. Grey background identifies the ‘normal’ contrast sensitivity range

In patients treated with PMOS, mean CS with glare improved significantly post-treatment spatial frequencies 3, 6, 12, and 18 cpd (*p* ≤ 0.03), with a trend toward statistical significance favoring PMOS at 1.5 cpd (*p* = 0.06) (Fig. 1). After treatment with PMOS, the mean contrast sensitivity frequencies fell within the normative range across all spatial frequencies [19]. When comparing the mean change in CS with glare between the two treatment groups, PMOS-treated patients had significantly greater improvement in CS with glare at 6 cpd (0.20 log unit difference; *p* = 0.02), 12 cpd (0.20 log unit difference; *p* = 0.02), and 18 cpd (0.15 log unit difference; *p* = 0.04).

**Fig. 2:**
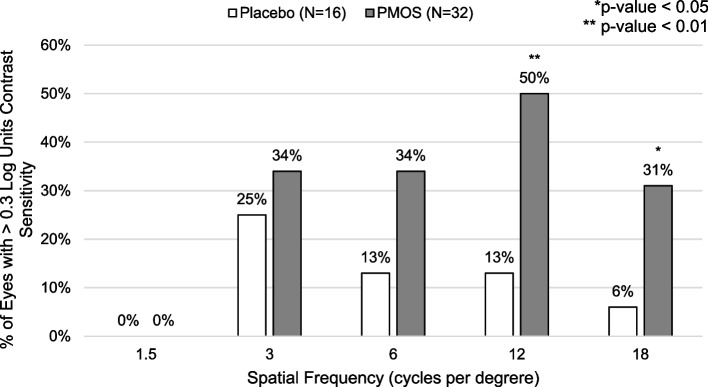
Percent of eyes with improvement in contrast sensitivity with glare of two or more patches (≥0.3 log units) by treatment group

The percent of eyes showing a 0.3 log units or greater increase (i.e., 50% improvement) in CS with glare was greater with PMOS treatment than placebo at 12 cpd (50% versus 13%, *p* < 0.01) and 18 cpd (31% versus 6%, *p* < 0.046). No eyes had a 0.3 log unit (two patches) or greater improvement in CS with glare at 1.5 cpd (Fig. 2).

Prior to treatment, mean contrast sensitivities without glare were not significantly different between treatment groups. In subjects treated with PMOS, mean CS without glare improved considerably at all frequencies tested (*p* < 0.05). In placebo-treated patients, there was an improvement in mean CS only at 3 cpd (*p* = 0.04). When comparing the mean change in CS without glare between the two treatment groups, PMOS-treated patients had a significantly greater improvement in CS without glare at 12 cpd (0.15 log unit, *p* = 0.021). No eyes experienced a ≥ 0.3 log unit (two patches) or greater improvement in CS without glare at 1.5 cpd.

### Visual Acuity (VA)

Multiple VA measurements were assessed including photopic distance low contrast visual acuity (pLCVA), mesopic distance low contrast visual acuity (mLCVA), photopic distance high contrast visual acuity (pHCVA), and mesopic distance high contrast visual acuity (mHCVA). In placebo-treated patients, there was no appreciable improvement from pre-treatment to 2–3 hrs post-treatment (min, max, SD) except for in mLCVA (18 letters read (1, 32, ± 9.3) vs. 21 letters read (8, 31, ± 7.7), *p* = 0.013). In PMOS-treated patients, statistically significant improvements in numbers of letters read were seen from pre-treatment across all VA measurements (*p* < 0.0001 for each): 23 letters read (12, 33, ± 5.7) vs. 31 letters read (13, 48, ± 8.2) for mLCVA; 62 letters read (42, 73, ± 7.4) vs. 69 letters read (60, 76, ± 5.1) for mHCVA; 69 letters read (45, 80, ± 7.9) vs. 73 letters read (64, 80, ± 4.9) for pHCVA; and 24 letters read (12, 34, ± 6.0) vs. 31 letters read (16, 46, ± 7.9) for pLCVA. When comparing the magnitude of improvement in mLCVA between placebo- and PMOS-treated patients, improvement with PMOS was significantly greater (8 letters read (− 8, 27, ± 8.4) vs. 3 letters read (− 1, 14, ± 4.5), respectively, *p* = 0.035). The magnitude of improvement with PMOS was also significantly greater for pLCVA (7 letters read (− 2, 23, ± 6.6) vs. 1 letters read (− 8, 6, ± 3.6), *p* < 0.001), and mHCVA (7 letters read (− 4, 24, ± 6.8) vs. 1 letters read (− 9, 8, ± 5.0), *p* < 0.01).

**Fig. 3:**
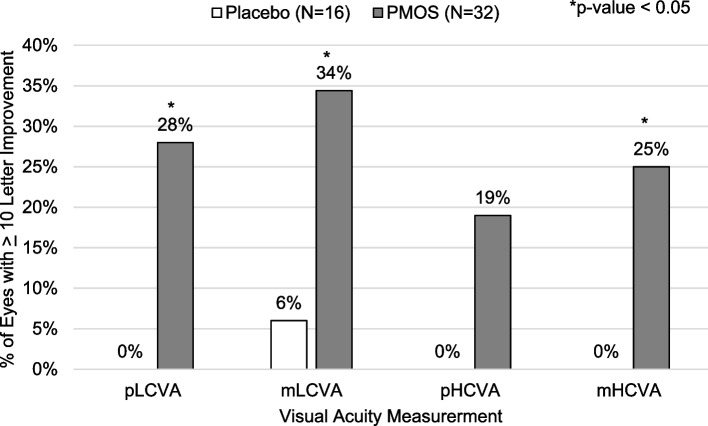
Percent of eyes with ≥ 10 letter improvement by visual acuity measurements by treatment group

**Fig. 4:**
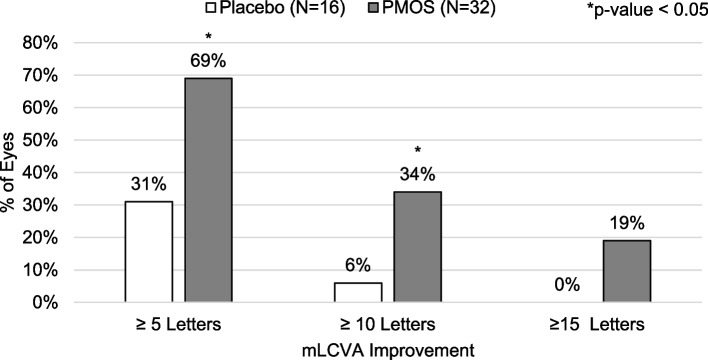
Percent of eyes with ≥ 5, ≥ 10, and ≥ 15 letter improvement in Mesopic Low Contrast Visual Acuity (mLCVA) by treatment group

**Fig. 5:**
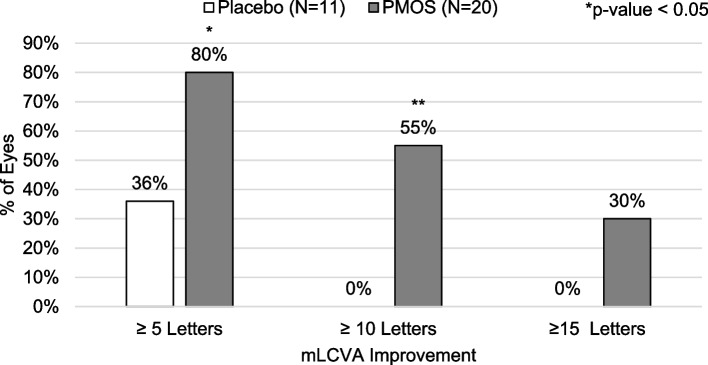
Percent of eyes (Baseline PD ≥ 6 mm) ≥ 5, ≥ 10, and ≥ 15 letter improvement in Mesopic Low Contrast Visual Acuity (MLCVA) by treatment group

Differences in mean change in VA between treatments were also reflected in a difference in the incidence of improvement of at least 10 letters between groups. No placebo-treated eyes registered a 10 letter or greater improvement in pLCVA, pHCVA, or mHCVA, though 6% of patients in the placebo group registered a 10 letter or greater improvement in mLCVA. Greater proportions of PMOS-treated eyes registered a 10 letter or greater improvement in pLCVA (28% vs. 0%, *p* < 0.02), mLCVA (34% vs. 6%, *p* < 0.03), pHCVA (19% vs. 0%, *p* = 0.16) and mHCVA (25% vs. 0, *p* < 0.03) (Fig. 3). In a post-hoc analysis, a statistically greater proportion of PMOS-treated eyes registered 5 letter (69% vs. 31%, *p* = 0.029) and 10 letter (34% vs. 6%, *p* = 0.04), with a trend toward statistical significance favoring PMOS at 15 letter (19% vs. 0%, *p* = 0.16), improvement in mLCVA compared to placebo (Fig. 4). To assess if a larger pre-treatment pupil diameter might contribute to a greater improvement in mLCVA, a post-hoc subgroup analysis in eyes with pre-treatment pupil diameter equal to or above 6 mm was performed and resulted in an even greater proportion of 5 letter (80% vs. 36%, *p* = 0.02) and 10 letter (55% vs. 0%, *p* = 0.004), with a trend toward statistical significance favoring PMOS at 15 letter (30% vs. 0%, *p* = 0.06) improvement in mLCVA improvement in eyes treated with PMOS relative to placebo (Fig. 5).

### Wavefront aberrometry

Two subjects in the PMOS group did not have post-dose wavefront measures collected due to equipment malfunction. Wavefront data were collected pre- and post-treatment on the remaining 14 PMOS-treated patients (28 eyes) and 8 placebo-treated patients (16 eyes). Mean pre-treatment WF diameter measures were not significantly different between placebo-treated eyes (5.7 mm) and PMOS-treated eyes (6.0 mm) (*p* = 0.26). Placebo-treated eyes showed no significant change in pupil diameter 2–3 hrs post-treatment (5.7 mm vs. 5.8 mm), while the mean pupil diameter of PMOS-treated eyes significantly decreased by 1.3 mm at 2–3 hrs after treatment (*p* < 0.0001). The difference in change across treatment arms was statistically significant (*p* < 0.0001).

Total wavefront root-mean-square (RMS) error is the standard deviation of all aberrations measured with a wavefront device, delineated in microns (μm), compared to an unaberrated ideal reference wavefront. Mean pre-treatment RMS error measures were not significantly different between placebo eyes (1.84 μm) and PMOS-treated eyes (1.45 μm) (*p* = 0.39). Placebo-treated eyes showed no significant change in RMS error 2–3 hrs post-treatment (*p* = 0.30), while the mean RMS error of PMOS-treated eyes significantly decreased by 0.47 μm post-treatment (*p* < 0.0001). The change from baseline was similar between treatment arms (between-group difference of 0.25 μm, *p* = 0.151).

Mean pre-treatment higher-order RMS error was significantly different between placebo eyes and PMOS eyes (0.62 μm vs. 0.36 μm, *p* < 0.002). Placebo-treated eyes showed no significant change in mean higher order RMS error post-treatment, while mean higher-order RMS error of PMOS-treated eyes significantly decreased by 0.14 μm (*p* < 0.0001). The difference in change between treatment arms (0.09 μm) was significant (*p* = 0.0176).

**Fig. 6:**
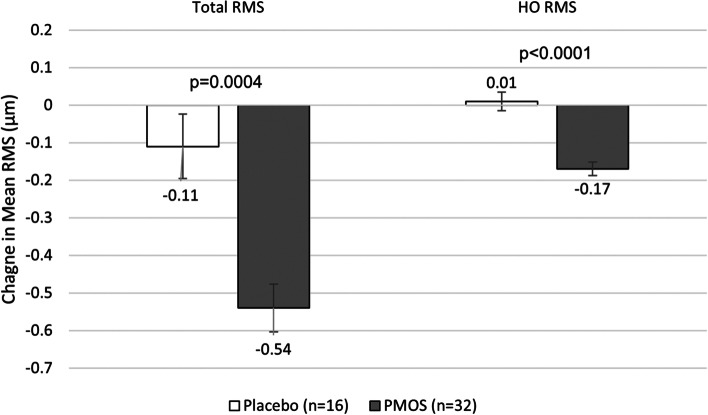
Improvement in mean total and higher-order wavefront aberrations by treatment group

To account for the correlation of the observations for the same subject, a more appropriate and stringent post-hoc analysis using a mixed-effects model with eye as a repeated measure was performed including an interaction term between treatment group and eye. The post-hoc analysis revealed statistical significance for both parameters in the difference between treatments. Mean total wavefront RMS error change between treatment arms was − 0.43 μm (*p* = 0.0004) and mean higher order RMS error change between treatment arms was − 0.18 μm (*p* < 0.0001) (Fig. 6).

### Subjective questionnaire

At 2–3 hrs post-treatment, a majority 69% (11/16) of subjects treated with PMOS rated their night vision as ‘improved,’ while 75% (6/8) of subjects treated with placebo rated their night vision as ‘the same’ as prior to treatment, and only 25% rated their night vision as ‘improved’. This difference in the percentage of PMOS and placebo subjects rating their vision as improved (69% vs. 25%) was significantly different (*p* = 0.049).

### Safety

No adverse events were reported during the study. In addition, no statistically significant differences were observed in mean HR or BP of patients at pre- or post-treatment for either treatment group. The mean change in IOP of PMOS-treated eyes from screening to 2–3 hrs post-treatment (− 1.8 mmHg) was significant (*p* < 0.0004); however, the change in PMOS-treated patients was not significantly different from the change in placebo-treated patients (between arm difference − 0.6 mmHg, *p* = 0.11).

Mean eye redness increased in both treatment groups. The change from baseline for the placebo group was minimal (+ 12.1 mm on a 100 mm visual analog scale; *p* = 0.0503), while the mean change in eye redness from baseline for the PMOS group was mild (+ 38.6 mm; *p* < 0.0001). Differences in mean change from baseline in eye redness for the two treatment groups were statistically significant (26.5 mm, *p* < 0.0004).

## Discussion

The purpose of the present study was to evaluate the efficacy of PMOS in reducing pupil diameter and improving contrast sensitivity with and without glare along with mesopic low and high contrast visual acuity in patients with severe night vision disturbances. We hypothesized that a single topical dose of PMOS would improve contrast sensitivity and mesopic visual function. Our objective findings and patient reported outcomes support this hypothesis, providing evidence that PMOS could serve as a pharmacologic treatment option for patients with ocular aberrations or ocular scatter that underlie DLD.

### Efficacy

PMOS-treated patients had significantly greater improvement in CS compared to placebo-treated subjects at 6, 12, and 18 cpd. Because CS at higher spatial frequencies correspond to the perception of smaller objects, PMOS could improve patients’ ability to recognize distant objects while performing activities in dim light conditions (e.g., driving at night) [20]. These results were found in the presence of glare, which has been shown to reduce nighttime driving performance by impairing motion sensitivity and mHCVA [21].

Reduction in best focus vision at low photopic and mesopic luminance is a consequence of higher retinal neural contrast threshold associated with reduced retinal illumination [22]. Visual acuity measures the smallest identifiable high-contrast target (i.e. in the higher frequency regions of the contrast sensitivity, 18 to 30 cpd) [20]. Although this study only assessed the efficacy of PMOS at 1.5, 3, 6, 12, and 18 cpd, it is possible that the benefits of PMOS could also extend to CS at 30 cpd. Further, PMOS-treated subjects demonstrated a statistical and clinically meaningful improvement in mLCVA over placebo. Additionally, post-hoc analysis revealed that PMOS may provide particular benefit to patients with larger baseline pupil diameter (6 mm or greater). Given the demonstrated improvement of contrast sensitivity and visual acuity in mesopic lighting conditions, this study provides evidence that PMOS could serve as a new treatment for DLD.

PMOS improved wavefront aberrations by decreasing both mean total RMS error and higher-order RMS error 2–3 hrs post-treatment. Xu et al. found that post-LASIK and keratoconus subjects with large amounts of higher-order aberrations had a reduction in starbursts with pupil size reduction to ≤3.0 mm [23]. After refractive surgery, brimonidine tartrate ophthalmic 0.15% solution, an alpha-2 agonist, has also been found to improve low contrast visual acuity, low contrast visual acuity with glare, and contrast sensitivity and ultimately decreasing night vision difficulty in some patients who underwent laser vision correction, however the miotic drug effect had dissipated at 1 month due to tachyphylaxis [24].

In contrast, PMOS, which reduces pupil diameter up to 36 hrs and shows no evidence of tachyphylaxis, could potentially improve functional vision in many patients with aberrated and/or scarred corneas, including patients with night myopia, multifocal or extended depth of focus IOLs, status post-refractive surgery, as well as other conditions with irregular corneal astigmatism such as keratoconus and corneal nodules. Even patients with cortical cataracts, where a smaller pupil would mitigate scattering of light from the periphery of the lens, could potentially have improved vision with PMOS [22].

### Safety

PMOS demonstrated a favorable overall safety profile. There were no adverse events or serious adverse events in the single-dose study, including no headaches, browaches, retinal tears, retinal detachments or vitreofoveal traction. Additionally, there were no systemic side effects. Mild to moderate hyperemia, a pharmacologic effect of PMOS as an alpha-1 adrenergic antagonist, was seen in some patients (18.8%). Although not studied in this trial, one-time use of over-the-counter vasoconstrictors or a nightly dosing regimen could minimize unwanted redness.

PMOS decreased IOP by 1.8 mmHg from a baseline of 13.8 mmHg which may have some clinical value given the benefit of reducing IOP by 1 mmHg [25]. Mean IOP was still in the normal range after treatment with PMOS. The observed decrease of IOP in normotensive patients is consistent with prior data, in which treatment with PMOS led to a decrease in IOP for patients with baseline IOP < 24 mmHg [26].

### Limitations

This study has several important limitations. First, the small sample size in this exploratory single center study precluded meaningful subgroup analysis, such as the relative impact on subjects with previous refractive surgery, keratoconus and other conditions associated with DLD. Future larger clinical trials will be needed to further characterize the efficacy of PMOS for DLD and determine whether efficacy varies by patient factors (e.g., iris color, mesopic pupil diameter). Second, measurements after baseline only occurred at one time point, limiting any analysis of improvement trends in visual function over time. Future studies should evaluate participants at additional time points to assess durability and neuroadaptation and also perform subset analyses to compare how DLD patients with specific underlying etiologies (e.g., keratoconus, myopic LASIK) or threshold higher-order aberrations at a given pupil diameter respond to treatment. Third, testing in this study was conducted in a controlled clinical environment. Future research to assess real-world conditions, such as night driving, could be useful to explore.

Prior studies have documented the inadequacy and reliability of visual acuity measurements, including ‘ceiling’ and ‘floor’ effects [27]. The floor effect, in which subjects do not read any contrast sensitivity patches, was present at high spatial frequencies (12 cpd, 18 cpd) in this study. Methods to more closely estimate the ‘floor’ include assigning a value of 0.3 log CS below the lowest CS score for patients with zero patches seen or assigning half of the lowest log CS value for patients with zero patches seen. However, these metrics, if applied, could overestimate the group means [28]. Emerging literature has suggested that based on the ‘ceiling’ and ‘floor’ effects, certain contrast sensitivity tests may be better for detecting subtle changes in normal, near-normal, or post-operative eyes [29]. Future studies should incorporate such tests to enhance the reliability of the findings.

## Conclusion

PMOS could be a viable and safe therapeutic option for DLD patients with various eye conditions that cause photic phenomena and decreased mesopic vision. Improvements in contrast sensitivity, visual acuity, patient-reported outcomes and decreases in wavefront aberrations all contribute to evidence of the potential benefits of PMOS for patients with DLD. Looking at regulatory approval pathways for a new DLD indication, the use of mLCVA versus contrast sensitivity as a primary endpoint would be more applicable and more standard for clinical trials.

### Future directions

In light of the favorable safety profile of PMOS demonstrated in this and other studies, combined with objective and subjective efficacy shown in this study in DLD patients, further later-stage studies are warranted [26, 30]. The LYNX-1 study (NCT04638660) is a registration Phase 3, multi-center, randomized, placebo-controlled, double-masked clinical trial targeting 140 adult subjects with DLD.

## Data Availability

The datasets used and/or analyzed during the current study are available from the corresponding author upon reasonable request.

## Abbreviations

BP: Blood pressure
CCLRU: Cornea and Contact Lens Research Unit
CI: Confidence interval
cpd: Cycles per degree
CS: Contrast sensitivity
DLD: Dim light vision disturbance
HR: Heart rate
IOL: Intraocular lenses
LASIK: Laser-assisted in situ keratomileusis
LCVA: Low contrast visual acuity
mHCVA: Mesopic distance high contrast visual acuity
mLCVA: Mesopic distance low contrast visual acuity
mm: Millimeters
pHCVA: Photopic distance high contrast visual acuity
pLCVA: Photopic distance low contrast visual acuity
PMOS: 1% Phentolamine Mesylate Ophthalmic Solution
PRK: Photorefractive keratectomy
RK: Radial keratotomy
RMS: Root mean square
WF: Wavefront
